# Prevalence, Severity, and Clinical Course of Atopic Dermatitis in Japan: A Web‐Based Questionnaire Survey

**DOI:** 10.1111/1346-8138.70329

**Published:** 2026-05-25

**Authors:** Takeshi Nakahara, Sakae Kaneko, Norito Katoh, Maki Ozawa, Hiroyuki Kanoh, Yutaka Hatano, Yuko Watanabe, Yasuyuki Sumikawa, Daisuke Onozuka, Akio Tanaka, Yoko Kataoka, Hiroyuki Murota

**Affiliations:** ^1^ Department of Dermatology Graduate School of Medical Sciences, Kyushu University Fukuoka Japan; ^2^ Department of Dermatology Masuda Red Cross Hospital Masuda Japan; ^3^ Department for Medical Innovation and Translational Medical Science Kyoto Prefectural University of Medicine Graduate School of Medical Science Kyoto Japan; ^4^ Toshoguekimae Dermatology Clinic Sendai Japan; ^5^ Department of Dermatology Gifu Municipal Hospital Gifu Japan; ^6^ Department of Dermatology Faculty of Medicine, Oita University Yufu Japan; ^7^ Watanabe Dermatology and Plastic Surgery Clinic Ueda Japan; ^8^ Dermatology & Allergy CLINIC Hokutoukai Medical Corporation Sapporo Japan; ^9^ Department of Post‐Infectious Diseases Therapeutics, Graduate School of Medicine The University of Osaka Osaka Japan; ^10^ Department of Dermatology Graduate School of Biomedical and Health Sciences, Hiroshima University Hiroshima Japan; ^11^ Department of Dermatology Osaka Habikino Medical Center Osaka Japan; ^12^ Department of Dermatology Nagasaki University Graduate School of Biomedical Sciences Nagasaki Japan

**Keywords:** atopic dermatitis, clinical course, prevalence, severity, web‐based questionnaire survey

## Abstract

Atopic dermatitis is a chronic inflammatory skin disease that commonly begins in childhood but exhibits heterogeneous long‐term clinical courses. We conducted a cross‐sectional questionnaire survey among dermatologists to evaluate age‐specific prevalence, remission proportion, severity, and disease course of atopic dermatitis in Japan. Data from 7777 individuals, including respondents and their family members, were analyzed. The prevalence of atopic dermatitis peaked at 5–9 years of age and gradually declined with age, although a substantial proportion of affected individuals remained in adulthood. Overall, prevalence was higher in males than in females, with a significant difference observed in the youngest age group. The proportion of individuals with a remitted disease course increased with age and plateaued after approximately 40 years. Remission proportions were higher in females than in males overall, although age‐specific differences were not consistently significant. Most cases were almost clear or mild; however, moderate‐to‐severe disease was observed in a subset of individuals across age groups, without marked sex differences. Regarding disease course, remission after childhood onset was the most common pattern, followed by relapse, persistence, and adult‐onset disease. Remission‐type courses were more frequent in females, whereas persistent and relapsing patterns were more common in males. These findings suggest that atopic dermatitis persists into adulthood in a substantial proportion of individuals and follows diverse clinical trajectories across the lifespan.

## 1 Introduction

Atopic dermatitis (AD) is a representative chronic inflammatory skin disease that commonly develops in childhood [1, 2]. However, recent studies have highlighted the presence of adult‐onset cases and relapses, indicating that the natural course of AD is not necessarily straightforward [3, 4]. In addition, the emergence of novel therapies, including biologics and Janus kinase (JAK) inhibitors, has increased interest in the disease concept and long‐term prognosis of AD. Regarding the epidemiology of AD, numerous studies have reported the prevalence in children [5, 6, 7, 8, 9, 10]; however, large‐scale data covering a wide age range, including adults, on prevalence, remission rates, severity distribution, and natural course remain insufficient. Particularly, studies simultaneously evaluating these parameters across different age groups and sexes remain limited.

In this study, we conducted a questionnaire survey targeting dermatologists and analyzed data from both respondents and their family members to evaluate the prevalence, remission rates, severity, and clinical course of AD. The aim of this study was to clarify the characteristics of AD prevalence and its clinical course across a broad age range.

## 2 Methods

### 2.1 Study Design and Participants

This study was a cross‐sectional web‐based questionnaire survey conducted by the AD Expert Committee of the Japanese Society for Cutaneous Immunology and Allergy. Data were collected between December 2025 and February 2026. The survey was approved by the Ethics Committee of Kyushu University (25029), and participation was voluntary. Responses were collected only from individuals who provided informed consent electronically before answering the questionnaire. Dermatologists were invited to participate, and if more than one dermatologist was present within the same family, only one representative was asked to respond. Invitations to participate in the survey were sent to 10 842 dermatologists, and responses were obtained from 1732 individuals, yielding a response rate of about 16%. Information regarding both respondents and their family members was collected.

### 2.2 Survey Population

The questionnaire collected information on the respondent and his or her family members. For the analyses of prevalence, participants included the respondent, spouse, father, mother, and children who were alive at the time of the survey. For the analyses of current disease severity, the target population was restricted to the respondent and family members currently living with the respondent. For the analysis of disease course, only respondents themselves were included.

### 2.3 Questionnaire Items

For the respondent, spouse, father, mother, and children, the following items were collected: age group, sex, current presence of AD, and past history of AD without current symptoms or treatment. Age was categorized into nine groups: 0–4, 5–9, 10–19, 20–29, 30–39, 40–49, 50–59, 60–69, and ≥ 70 years. Sex was recorded as male or female. For the respondent and currently cohabiting family members, current AD severity was assessed in individuals with current AD using a 4‐point Investigator's Global Assessment (IGA)‐based scale: almost clear, mild, moderate, and severe. For respondents who had current or past AD, the clinical course of AD was assessed by selecting the single pattern that best described their disease course: (1) childhood onset with remission, (2) childhood onset with persistence, (3) childhood onset followed by improvement and later relapse, or (4) onset after adolescence (Table S1).

### 2.4 Statistical Analysis

Sex differences in proportions were analyzed using a two‐sample test of proportions. All statistical analyses were performed using Stata 19.0 (Stata Corp, College Station, Texas). The significance level for all tests was *p* < 0.05 (two‐sided).

## 3 Results

### 3.1 Characteristics of the Study Population

A total of 1732 responses were obtained, and 1690 individuals with complete age and sex data were included in the analysis (Table 1). The total study population, including respondents and their family members, comprised 7982 individuals, of whom 7777 with complete demographic data were analyzed (Table 2).

**TABLE 1:** Age and sex distribution of all respondents.

Age	Male (*n*)	Female (*n*)	Total (*n*)
20–29	19	23	42
30–39	97	142	239
40–49	145	238	383
50–59	253	246	499
60–69	217	165	382
≥ 70	103	42	145
Total	834	856	1690

**TABLE 2:** Number of analyzed individuals (respondents and family members with available age and sex data).

Age	Male (*n*)	Female (*n*)	Total (*n*)
0–4	92	99	191
5–9	146	142	288
10–19	425	367	792
20–29	328	382	710
30–39	417	442	859
40–49	406	513	919
50–59	466	536	1002
60–69	538	571	1109
≥ 70	964	943	1907
Total	3782	3995	7777

### 3.2 Age‐Specific Prevalence of AD

**FIGURE 1:**
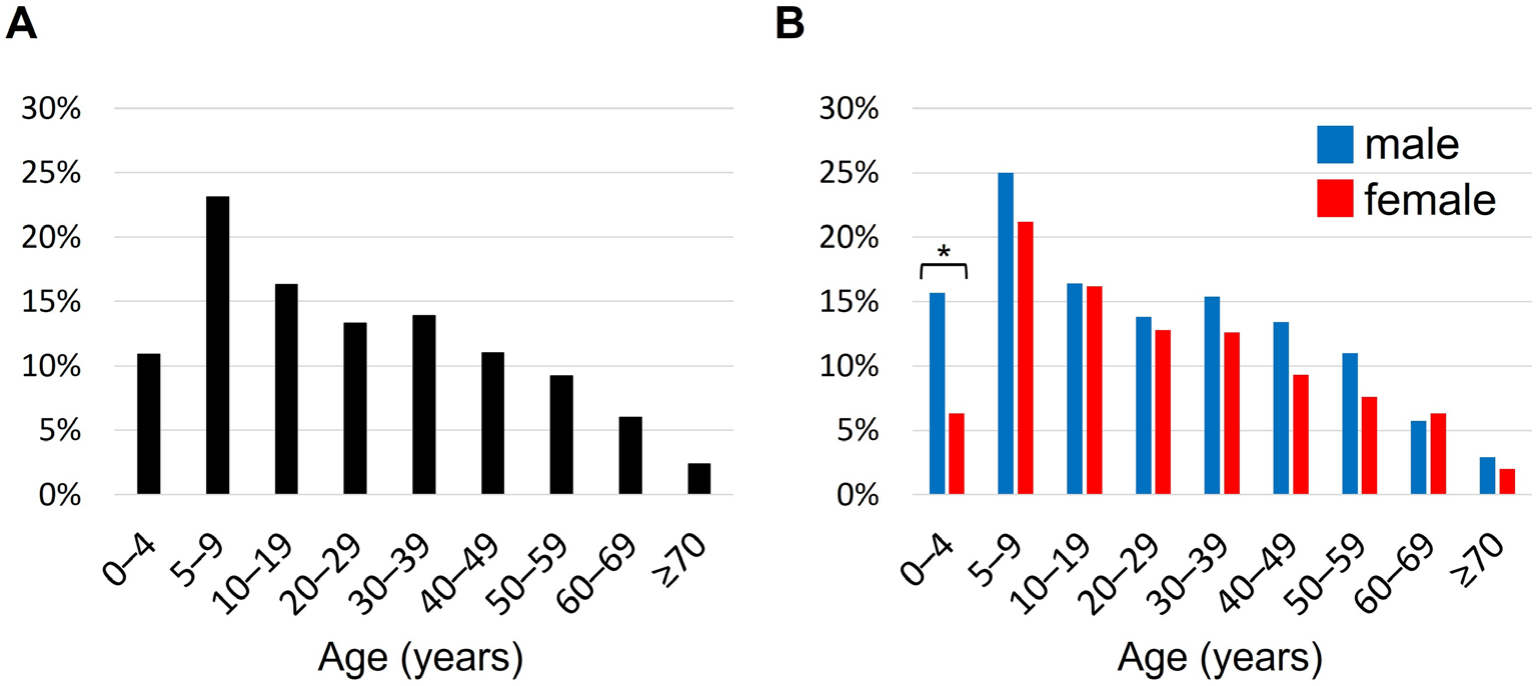
Age‐specific prevalence of AD in the study population (dermatologists and their family members). (A) Overall age‐specific prevalence of AD in the total population. (B) Age‐specific prevalence of AD in males and females. Sex differences in proportions were analyzed using a two‐sample test of proportions. A significant difference was observed in the 0–4‐year age group (*p < 0.05, two‐sided).

The age‐specific prevalence of AD showed a peak in childhood, particularly in the 5–9‐year age group, and gradually declined with increasing age thereafter. In the overall population, the prevalence was 10.9% in those aged 0–4 years, increased to 23.1% at 5–9 years, and then decreased to 16.3% at 10–19 years, 13.3% at 20–29 years, 13.9% at 30–39 years, 11.0% at 40–49 years, 9.2% at 50–59 years, 6.0% at 60–69 years, and 2.4% at ≥ 70 years (Figure 1A). Despite this age‐related decline, a substantial proportion of adults continued to have AD. When analyzed by sex, the prevalence of AD was significantly higher in males than in females (*p* = 0.006). Age‐stratified analysis showed that the age‐related prevalence pattern was generally similar between sexes; however, a significant sex difference was observed only in the youngest age group (0–4 years), in which prevalence was higher in males than in females (*p* = 0.004). Differences in other age groups were small and not statistically significant (Figure 1B).

### 3.3 Age‐Specific Remission Proportion

**FIGURE 2:**
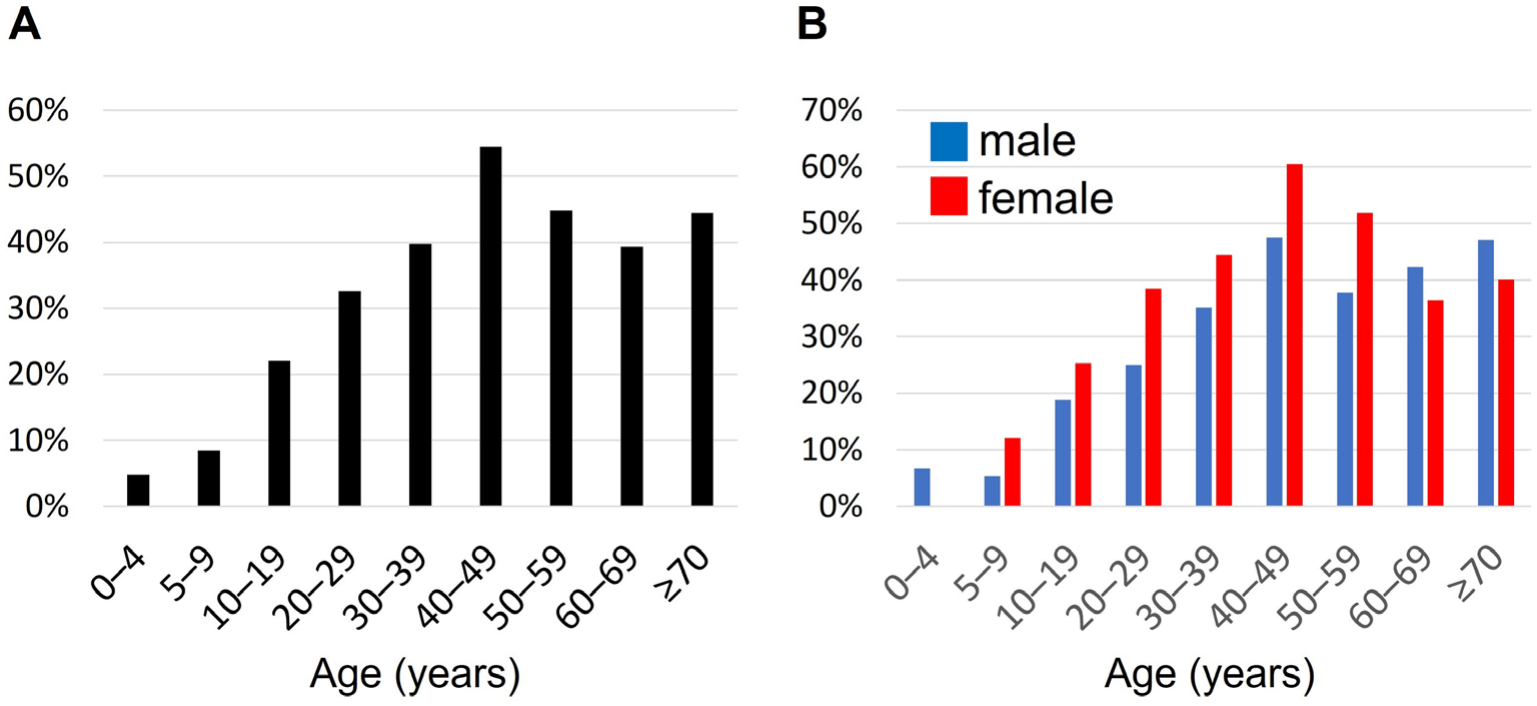
Age‐specific remission proportion of AD. Remission proportion was defined as the proportion of individuals with a past history of AD but no current disease among those with either current AD or a past history of AD within each age group. (A) Overall age‐specific remission proportion in the total population. (B) Remission proportion stratified by sex. Sex differences were analyzed using a two‐sample test of proportions; however, no statistically significant differences were observed across age groups (two‐sided, *p* ≥ 0.05).

The proportion of individuals with a remitted disease course increased with age and plateaued after approximately 40 years. In the overall population, the proportion increased from 4.8% in those aged 0–4 years to 8.5% at 5–9 years, 22% at 10–19 years, 32.6% at 20–29 years, and 39.8% at 30–39 years, reaching a peak of 54.5% at 40–49 years, followed by 44.8% at 50–59 years, 39.3% at 60–69 years, and 44.4% at ≥ 70 years (Figure 2A). Importantly, this “remission proportion” does not represent the rate of remission occurring at each age. Rather, it reflects the proportion of individuals within each age group who had experienced remission during their disease course among those with current or past AD. Specifically, the remission proportion was calculated as the number of individuals with a past history of AD but no current disease divided by the total number of individuals with either current AD or a past history of AD within each age group. Thus, the observed increase with age should be interpreted as a cumulative pattern of disease course within each age group. When analyzed by sex, remission proportions were significantly higher in females than in males overall (*p* = 0.002). Although females tended to have slightly higher remission proportions across several age groups, these differences were not statistically significant within individual age categories (Figure 2B).

### 3.4 Age‐Specific Severity of AD

**FIGURE 3:**
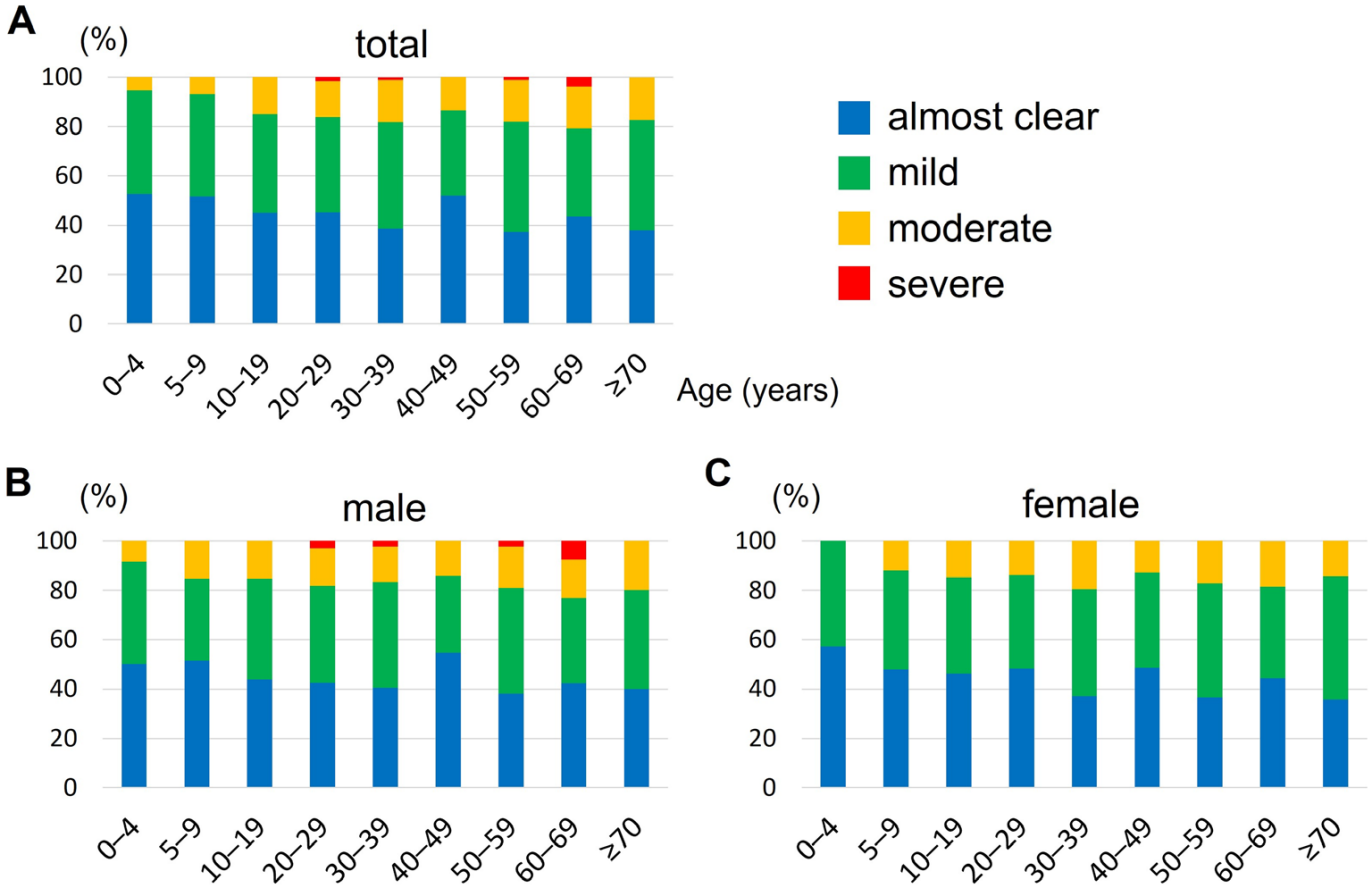
Age‐specific distribution of disease severity in AD. Disease severity was classified as almost clear, mild, moderate, and severe. (A) Total population. (B) Males. (C) Females. When moderate‐to‐severe cases were combined, sex differences were analyzed using a two‐sample test of proportions, and no significant differences were observed.

Across all age groups, most individuals with current AD were classified as having almost clear or mild disease. In the overall population, these categories accounted for the majority of cases in all age groups, whereas moderate disease was observed in a smaller but consistent proportion of individuals, and severe disease was rare (Figure 3A). The proportion of moderate disease was lower in younger children, increased from adolescence to adulthood, and remained relatively stable thereafter. Severe disease was uncommon in all age groups and was observed only in males in a limited number of age groups (Figure 3B). When stratified by sex, no consistent or marked differences were observed between males and females in the overall distribution of disease severity (Figure 3B,C).

### 3.5 Clinical Course of AD

**FIGURE 4:**
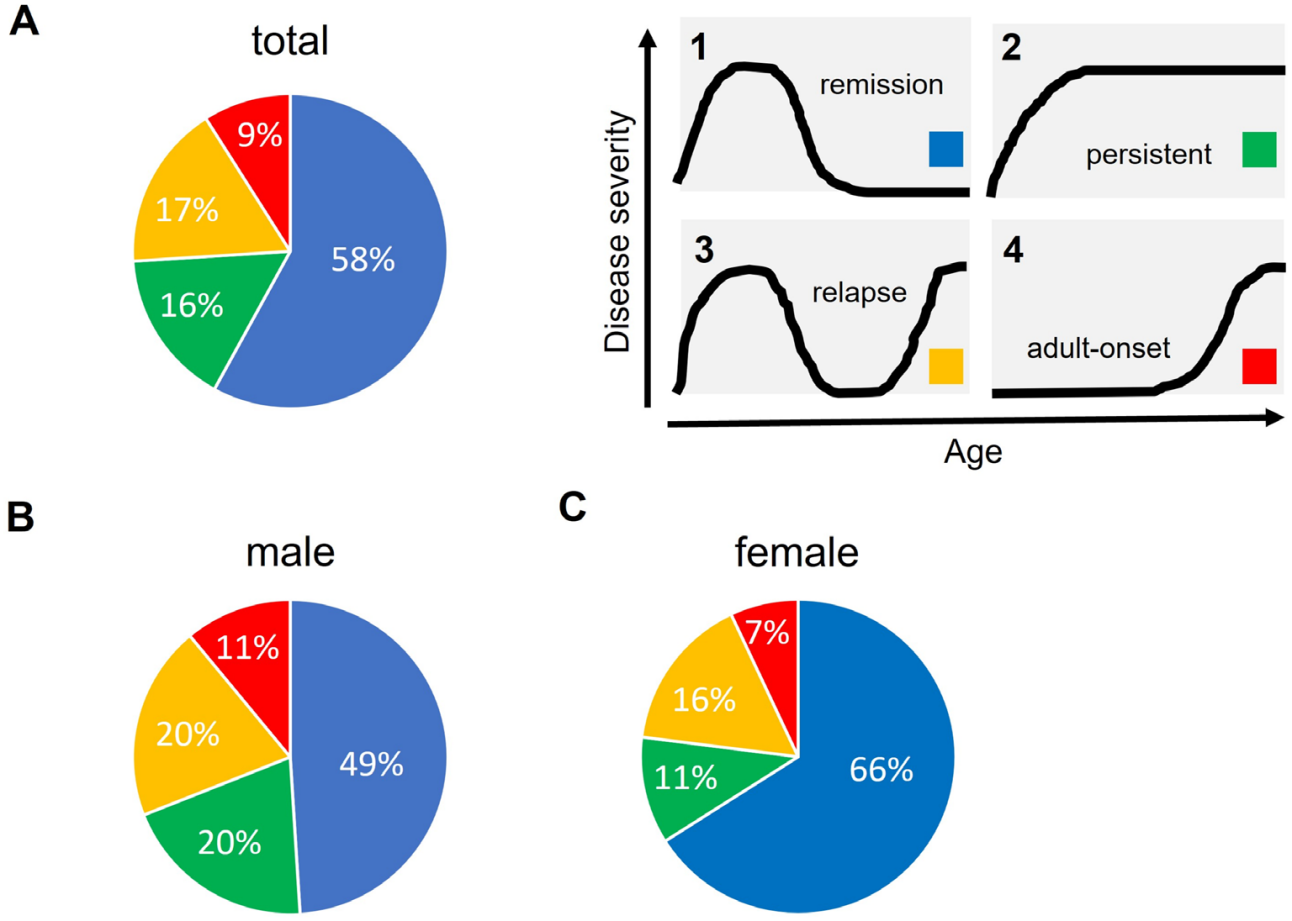
Distribution of clinical course patterns in AD. Clinical course was categorized as remission, persistent disease, relapse, and adult‐onset disease. (A) Total population. (B) Males. (C) Females. Sex differences in proportions were analyzed using a two‐sample test of proportions. A significant difference was observed for the remission pattern, which was more frequent in females than in males (*p < 0.05, two‐sided).

The distribution of clinical course patterns is shown in Figure 4. This analysis was based on responses from the dermatologists themselves. Among individuals with current or past AD, remission after childhood onset was the most common disease course, accounting for approximately 57.9% of cases. This was followed by relapse after initial improvement (17.5%), persistent disease from childhood (15.7%), and adult‐onset disease (8.9%) (Figure 4A). When stratified by sex, remission was associated with sex (*p* < 0.001). Remission after childhood onset was more frequent in females (66.0%) than in males (49.0%). In contrast, persistent disease and relapse tended to be more common in males (20.0% and 20.0%, respectively) than in females (12.0% and 16.0%, respectively). Adult‐onset AD was observed in both sexes, with a slightly higher proportion in males (11.0%) than in females (7.0%) (Figure 4B,C). Overall, while remission was the predominant disease course, a substantial proportion of individuals exhibited non‐remitting patterns, including persistence and relapse, highlighting the heterogeneous nature of AD over time.

## 4 Discussion

In this study, we comprehensively evaluated age‐specific prevalence, remission proportion, severity distribution, and clinical course of AD using a large‐scale questionnaire survey of dermatologists and their family members. The prevalence of AD peaked in childhood, particularly at 5–9 years of age, and declined with age; however, a substantial proportion of adults remained affected. The proportion of individuals with a remitted disease course increased with age, although this should be interpreted as a cumulative pattern within each age group rather than within an age‐specific remission rate.

The prevalence of AD in children has been widely reported worldwide, with the ISAAC study demonstrating substantial geographic variation [5, 6]. In Japan, the prevalence among school‐aged children has been reported to be approximately 10%–20%, consistent with our findings [7]. While previous studies have largely focused on pediatric populations, our study, which included a broad age range and a predominantly adult population, provides valuable insights into the age‐spanning characteristics of AD. The persistence of AD prevalence in adults observed here supports the growing recognition that AD is not confined to childhood but may persist into or newly develop in adulthood [3, 4]. Taken together, these results emphasize the importance of considering AD as a lifelong disease with heterogeneous clinical trajectories.

Regarding sex differences, the higher prevalence of AD in males and the greater remission proportion observed in females may suggest sex‐related differences in disease persistence and resolution. These disparities may reflect underlying biological differences in immune regulation and hormonal influences, which have been implicated in sex‐specific susceptibility and clinical course in AD [11]. Behavioral factors, including sex‐related differences in treatment awareness and adherence, may also contribute to these observed disparities. Previous epidemiological studies have generally reported a male predominance in early childhood and a female predominance after adolescence in AD prevalence. In contrast, our study demonstrated a persistent male predominance in overall prevalence, while females more frequently exhibited a remittent disease course. These discrepancies may reflect differences in study population, ethnicity, survey methodology, or healthcare‐seeking behavior. With respect to disease severity, most cases were classified as almost clear or mild across all age groups, while moderate disease accounted for a consistent proportion, and severe disease was rare. Although severe cases were observed only in males, the number was small and should be interpreted with caution. Overall, AD in the general population appears to be predominantly mild, with only a small subset of patients experiencing more severe disease. Analysis of disease course revealed that remission after childhood onset was the most common pattern, followed by relapse, persistence, and adult‐onset disease. These findings highlight the heterogeneous nature of AD and are consistent with previous reports demonstrating diverse disease trajectories [2, 3]. The presence of adult‐onset cases further supports the concept that AD is not exclusively a childhood disease.

A major strength of this study is that the participants were dermatologists and their family members, which likely enhanced the accuracy of diagnosis, severity assessment, and reporting of disease course compared with surveys conducted in the general population. Medically informed respondents may provide more reliable information regarding disease history and severity. However, several limitations should be noted. First, the study population differs from the general population, and variations in healthcare access and treatment practices may have influenced the observed prevalence and disease course. Second, mild or minimal skin symptoms may have been more readily recognized and reported as AD, potentially leading to overestimation of mild cases. Third, the response rate to the questionnaire was relatively low, which may have introduced selection bias and influenced the results. Finally, as this was a cross‐sectional study, temporal changes in individual disease trajectories could not be directly assessed.

In conclusion, AD peaks in childhood but persists into adulthood in a substantial proportion of individuals and exhibits diverse clinical courses. Our findings provide important epidemiological insights into AD across a wide age range, particularly in adult populations, and contribute to a better understanding of its long‐term clinical course. Repeated surveys conducted over time may further help to capture temporal trends and clarify the impact of evolving treatment strategies on the epidemiology and disease course of AD.

## Ethics Statement

This study was approved by the Ethics Committee of Kyushu University (approval no. 25029). All participants provided informed consent prior to participating in the study.

## Conflicts of Interest

Takeshi Nakahara has received consulting fees and/or speaker honoraria from Torii Pharmaceutical Co. Ltd., Maruho Co. Ltd., Sanofi, AbbVie, Eli Lilly Japan, Sun Pharma, and Otsuka Pharmaceutical Co. Ltd. Norito Katoh has received honoraria as a speaker/consultant for Sanofi, Maruho, Abbvie, Eli Lilly Japan, Taiho Pharmaceutical, Pfizer, Mitsubishi Tanabe Pharma, Jansen Pharma, Kyowa Kirin, Celgene Japan, Torii Pharmaceutical, Novartis Pharma, and Otsuka Pharmaceutical, and has received grants as an investigator from Mitsubishi Tanabe Pharma, Torii Pharmaceutical, Maruho, Sun Pharma, Boehringer Ingelheim Japan, and Leo Pharma. Norito Katoh is an Editorial Board member of *Journal of Dermatology* and a coauthor of this article. To minimize bias, he was excluded from all editorial decision‐making related to the acceptance of this article for publication. Maki Ozawa has received consulting fees and/or speaker honoraria from Torii Pharmaceutical Co. Ltd., Maruho Co. Ltd., Sanofi, AbbVie, Eli Lilly Japan, Mitsubishi Tanabe Pharma, Leo Pharma and Otsuka Pharmaceutical Co. Ltd. Yutaka Hatano has received speaker honoraria from Torii Pharmaceutical Co. Ltd., Sanofi K.K., Otsuka Pharmaceutical Co. Ltd., and Regeneron Pharmaceuticals Inc. Yasuyuki Sumikawa has received honoraria as speaker/consultant for Sanofi, Maruho, Eli Lilly Japan, Pfizer, Novartis Pharma, and Kao. Akio Tanaka has received consulting fees and/or speaker honoraria from AbbVie GK, Eli Lilly Japan, Tanabe Pharma Corporation, Pfizer Japan Inc., Sanofi K.K., Torii Pharmaceutical Co. Ltd., Otsuka Pharmaceutical Co. Ltd., and Maruho Co. Ltd., and research grants not related to the submitted work from Maruho Co. Ltd. Yoko Kataoka has received speaker honoraria from AbbVie, Sanofi, Maruho, Pfizer and research grants from Sanofi, AbbVie, Amgen, Eli Lilly Japan, LEO Pharma, Maruho, Otsuka Pharmaceutical, Taiho Pharmaceutical and Pfizer Japan. Hiroyuki Murota was speaker and/or consultant and/or Investigator of Leo Pharma, Lilly, Novartis Pharma, AbbVie, Sanofi, Pfizer, Maruho Co. Ltd., Torii pharma, Otsuka pharma.

## Supporting information


**Table S1:** jde70329‐sup‐0001‐TableS1.docx.

## Data Availability

The data that support the findings of this study are available from the corresponding author upon reasonable request.
