# Supplementary material for: Prevalence, Severity, and Clinical Course of Atopic Dermatitis in Japan: A Web‐Based Questionnaire Survey
Source: J Dermatol. 2026 May 25;53(7):990–5. doi: 10.1111/1346-8138.70329 (PMC13341073; doi:10.1111/1346-8138.70329)
Supplement: Supplementary file 1 — Table S1: jde70329‐sup‐0001‐TableS1.docx. [file JDE-53-990-s001.docx]

**Table S1**

**Atopic Dermatitis Survey Questionnaire**

This survey is conducted by the Atopic Dermatitis Expert Committee of the Japanese Society of Cutaneous Immunology and Allergy (approved by the Kyushu University Ethics Committee). Participation is voluntary, and your cooperation is greatly appreciated. The collected data will be used solely for this survey.
If multiple family members are dermatologists, please respond only once per family, by the representative family member.

**Do you agree to participate in this survey?**

1. Yes, I agree
2. No, I do not agree

**1. Please answer Questions 1-1–1-4 regarding yourself, your spouse, parents, and children.**

(Please respond only for family members who are currently alive.)

**1-1. Age**

(Select the appropriate age category by number 1–9)

1. 0–4 years
2. 5–9 years
3. 10–19 years
4. 20–29 years
5. 30–39 years
6. 40–49 years
7. 50–59 years
8. 60–69 years
9. ≥70 years

**1-2. Sex**

1. Male
2. Female

**1-3. Current presence of atopic dermatitis**

1. Yes
2. No
3. Unknown

**1-4. Past history of atopic dermatitis**(defined as no current symptoms or treatment)

1. Yes
2. No
3. Unknown

**2. Please answer regarding yourself and family members currently living with you.**

**If currently affected by atopic dermatitis, select the present disease severity.**

Severity is based on the Investigator’s Global Assessment (IGA) as follows:
1 = Almost clear
2 = Mild
3 = Moderate
4 = Severe

1. Almost clear
2. Mild
3. Moderate
4. Severe
5. Unknown

**3. If you currently have or previously had atopic dermatitis, please answer the following question about yourself.**

**Select the clinical course that most closely matches your atopic dermatitis history.**

1. Childhood-onset, resolved
2. Childhood-onset, persistent
3. Childhood-onset, improved temporarily, then relapsed
4. Adolescent/adult-onset

**Thank you for your participation.**
